# Integrating Overlapping Structures and Background Information of Words Significantly Improves Biological Sequence Comparison

**DOI:** 10.1371/journal.pone.0026779

**Published:** 2011-11-10

**Authors:** Qi Dai, Lihua Li, Xiaoqing Liu, Yuhua Yao, Fukun Zhao, Michael Zhang

**Affiliations:** 1 College of Life Sciences, Zhejiang Sci-Tech University, Hangzhou, People's Republic of China; 2 Institute for Biomedical Engineering and Instrumentation, Hangzhou Dianzi University, Hangzhou, People's Republic of China; 3 School of Science, Hangzhou Dianzi University, Hangzhou, People's Republic of China; 4 Cold Spring Harbor Laboratory, Cold Spring Harbor, New York; University of Nottingham, United Kingdom

## Abstract

Word-based models have achieved promising results in sequence comparison. However, as the important statistical properties of words in biological sequence, how to use the overlapping structures and background information of the words to improve sequence comparison is still a problem. This paper proposed a new statistical method that integrates the overlapping structures and the background information of the words in biological sequences. To assess the effectiveness of this integration for sequence comparison, two sets of evaluation experiments were taken to test the proposed model. The first one, performed via receiver operating curve analysis, is the application of proposed method in discrimination between functionally related regulatory sequences and unrelated sequences, intron and exon. The second experiment is to evaluate the performance of the proposed method with f-measure for clustering Hepatitis E virus genotypes. It was demonstrated that the proposed method integrating the overlapping structures and the background information of words significantly improves biological sequence comparison and outperforms the existing models.

## Introduction

With the development of high-throughput sequencing technology, the rate of addition of new sequences to the databases increases continuously. However, such a collection of sequences does not by itself increase the scientist's understanding of the biology of organisms. Comparing a new sequence with the sequences of known functions is an effective way of assigning function to the new genes/proteins and understanding the biology of that organism from which the new sequence comes.

Owing to the importance of sequence comparison, numerous researches have been taken in past and obtained some effective tools for similarity search [1]–[8], evolutionary study [9]–[19], and classification [20]–[23]. The methods developed for sequence comparison can be categorized into two classes. One is alignment-based methods, in which a matrix of numbers that represents all possible alignments between two sequences is obtained with dynamic programming, and the highest set of sequential scores in the matrix defines an optimal alignment. Waterman (1995) and Durbin et al. (1998) provided comprehensive reviews about this method [24], [25]. But the search for optimal solutions using alignment-based method has problems in: (i) computational load with regard to large databases [2]; (ii) choice of the scoring schemes [26]. Therefore, the emergence of research into the second class, alignment-free method, is apparent and necessary to overcome critical limitations of alignment-based methods [2], [3], [5], [6], [12], [13].

Up to now, many efficient alignment-free methods have been proposed, but they are still in the early development compared with alignment-based measure [2], [5], [6], [26]–[36]. One of the most widely used alignment-free approaches is word-based model that meets the need for rapid sequence comparison. In this model, each sequence is first mapped into an 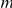-dimensional vector according to its 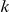-word frequencies, and sequence similarity can then be measured by distance measures, such as Euclidean distance [27], Mahalanobis distance [28], Kullback-Leibler discrepancy [29], [30] and Cosine distance [31]. When the 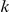-words occurring in biological sequence are estimative probabilities rather than the frequencies, they are more readily optimized by more complex models, such as Markov model [2], [33]–[35], mixed model [5], [6] and Bernoulli model [36]. These complex models could be considered to be the modification of traditional word-based models, in which several critical problems still exist in their development as described below.

First, little attention has been paid to the overlapping structures of the words in biological sequences [2], [5], [27]–[29], [31], [33], [34]. Overlapping occurrences of a word 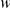 are the occurrences of the word 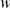 that overlaps the previous occurrence of the word 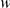. For instance, in the sequence ACGAATAATAAATAAGGCAATAAC, there are four occurrences of AATAA (starting at positions 4, 7, 11 and 19). But the occurrence of AATAA starting at the position 4 is different from the one starting at the position 19, because the form is composed of three overlapping occurrences of AATAA whereas the second one is composed of a unique occurrence. Because the overlapping structure of the words usually form conservative patterns in biological sequences that are strongly associated with genes [37], [38], the overlapping structures of the words should be taken into account when comparing two biological sequences.

Second, background information of the words has not been fully utilized in existing biological sequence comparison [27]–[29], [31], [33], [34], [36]. Mutations take place randomly at molecular level, and natural selections shape the direction of evolution. In order to highlight the contribution of selective evolution, random background from the simple counting result was proposed to build a composition vector (CV) and has been used with minor modification for phylogenetic studies of prokaryotes and viruses [33], [34]. Recently, Lu 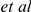. found some statistical problems associated with composition vector (CV) and proposed an improved composition vector (ICV) method based on a known word distribution [36]. However, due to the fact that the word distribution is usually unknown in most cases, and each biological sequence has its own word distribution, the ICV method is of limited use.

This paper proposed an efficient statistical method for sequence comparison. It takes into consideration the overlapping occurrences of the words and has the ability to adjust the background information of the words in biological sequences. The contents can be summarized as follows:

1. An efficient word-based statistical measure based on the statistical model proposed by Schbath [39] was proposed, which utilizes the Markov model to estimate the variance of word frequencies and decomposes the similarity score into a sum of similarities of the normalized word frequencies.
2. Extensive experiments were taken to evaluate the performance of proposed model in discrimination between (a) functionally related regulatory sequences and unrelated sequences, intron and exon, and (b) different HEV genotypes. A comparison of proposed method with existing alignment-based and alignment-free models was also taken to assess its superiority.

## Methods

### Word-based Statistical Models (WSM)

#### Background information of words

A biological sequence can be described as a succession of symbols, and a 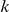-word is a series of 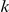 consecutive letters in the sequence. For a sequence 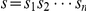, the count of a 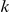-word 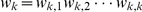, denoted by 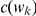, is the number of occurrence of the word 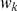 in the sequence 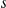. The position of an occurrence of the word 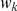 is defined by the position of its first letter 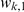. We define a random indicator 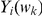 of an occurrence of 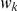 at position 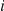, 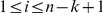, in 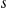 by
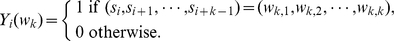
The occurrence frequency of the word 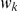 in the sequence 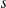 can be calculated with the random indicators of occurrence
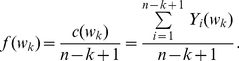
(1)


DNA and protein sequences have been realized to be a mixture of local regions that consist of compositional characteristics and pseudo-periodic sequence patterns. To utilize the background information of these local regions, we choose Markov model as a background model. It takes into consideration this ‘periodical’ behavior of the bio-signal by making use of transition probability matrix 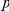 and initial state distribution 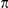.

Because 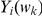 is a random Bernoulli variable, the probability 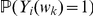 under the Markov model with order 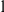 (M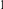) can be calculated by
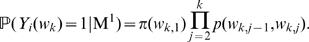
(2)For convenience, let 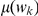 denote the probability of the word 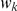 to appear at a given position in the sequence, and expectation of the 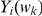 under the Markov model (M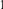) is 
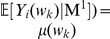
. With the expectation 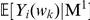, we can get the expectation of the word frequency 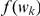 under the Markov model (M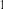)
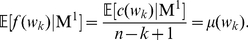
(3)


#### Overlapping structures of words

Occurrences of the same word may overlap, and these overlapped words usually form a conservative pattern that is strongly associated with conservative motif [38]. So it is valuable that the overlapping structures of the words are taken into consideration when comparing two biological sequences. Here, we measure the ability of a word to overlap itself with a overlapping indicator, 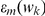 , defined as follows:
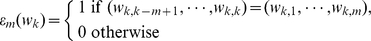
where 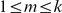. With the 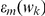, we can calculate the probability of observing two overlapping occurrences with 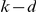 (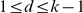) letters in common and two non-overlapping occurrences of the word 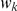 separated by 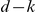 letters (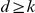) under the Markov model (M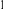) as follows:
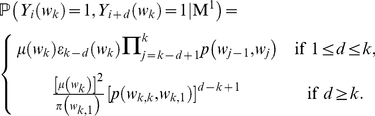
(4)Since the variables 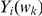 and 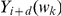 are not independent under the Markov model [39]–[41], their effects can be described by their covariance
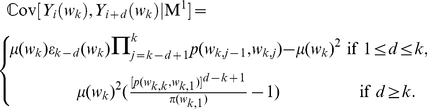
(5)With the above formulas, we can calculate the variance of the 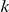-word frequency 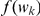 under the Markov model (M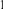)
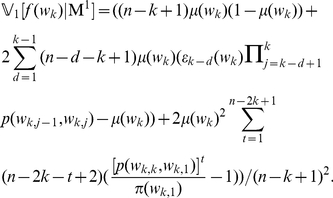
(6)What we have presented above is the 1-order Markov model, generalizations to high order can be deduced similarly.

#### Word statistical model

By incorporating the overlapping structures and the background information of the words in the existing statistical model, a novel word-based statistical model is proposed and denoted in a compact form
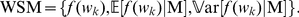
(7)in which the sequence information obtained through the statistical properties of the words was integrated with the overlapping structures and the background information of the words.

There are several distinctive features of this model. First, it emphasizes the structures of the words and indicates differences in terms of their contribution to the conservative patterns. Second, the influence of two overlapping occurrences of the word 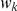 with 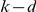 (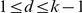) letters in common and two non-overlapping occurrences of the word 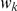 separated by 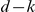 letters (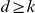) is considered. Finally, Markov model is chosen as the background model instead of Bernoulli model because each biological sequence should have its own word distribution.

### Parameter estimation

Since the model parameters are priori unknown, they have to be estimated based on the observed sequences. The accuracy of this estimation is an important issue to be considered, and the existing perturbation theory for Markov chains and hidden Markov models can allow us to assess the uncertainty in the Markov chain behavior given the uncertainty [42], [43]. In this paper, rather than assuming a known word distribution like [36], we estimate the model parameters with the maximum likelihood method [25] and replaces 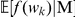 by the following estimator
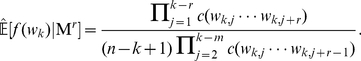
(8)As for the variance, there are several approaches to derive the asymptotic variance. According to the methods proposed by Schbath [39], we have
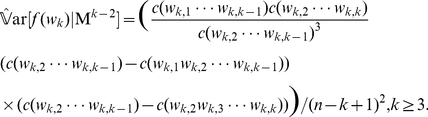
(9)However, in an application where 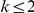, we derive the asymptotic variance under Markov model M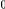 (Bernoulli model)
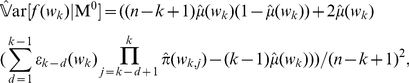
(10)where 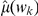 is the estimator of 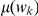, 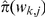 is the estimator of 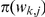.

### Statistical similarity measure

With the assumption of the uniform distribution (U), Lu [36] calculated the word expectation and variance, and defined the normalization function 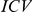 as:
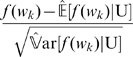
(11)where 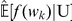 and 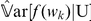 are the expectation and variance of the word frequency 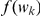. The normalization function 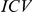 is necessary but not sufficient, because much effort of this method is to find better ways to utilize evolution information. In addition, the function 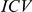 relies heavily on the word distribution. When the expectation based on background model is strongly associated with the 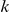-word frequencies, this function can carry more information, otherwise it will increase the noise accompanied by words with exceptional background frequencies.

For the probability distributions 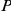 and 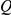 of a discrete random variable, the relative entropy (also called Kullback-Leibler divergence) of 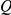 from 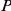 is defined as
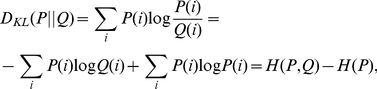
(12)where 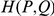 is the cross entropy of 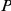 and 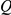, and 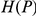 is the entropy of 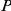. The relative entropy is the most important concept in both statistical biology and information theory. It has been deployed as non-distance similarity measures, such as 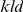 [29], [30] and 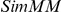 [2], to compare biological sequences.

A statistical measure between two proposed statistical models was proposed here based on the cross entropy 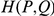 and Euclidean distance. It is denoted by 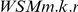 as follows:
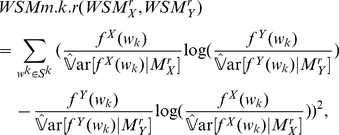
(13)where 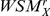 and 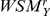 are two statistical models with Markov order 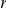 for two biological sequences 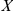 and 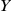, and the set 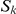 consists of all possible sequences of length 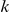 with symbol from the alphabet 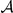. In the context of DNA sequences, 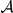 is {A,C,G,T}. It is noticed that the similarity measure 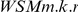 satisfies the identity and triangle, but it does not satisfies inequality conditions. So it is only a dissimilarity measure. Another point of interest about this similarity measure is its normalization function that can reduce the noise by ignoring the word expectation in its definition.

### Receiver operating curve and F-measure

*Receiver Operating Curve analysis*. Receiver operating curve (ROC) analysis has been widely used in signal detection and classification [44]. It is usually employed in binary classification of continuous data categorized as positive (1) or negative (0) cases. The classification accuracy can be measured by sensitivity and specificity, which are defined as
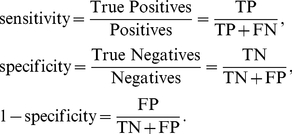
(14)


ROC curve is a graphical plot of sensitivity versus (1-specificity) for different threshold values. The area under a ROC curve (AUC) is an important value used to quantify the quality of a classification because it is a threshold independent performance measure and is closely related to the Wilcoxon signed-rank test [45]. A comprehensive discussion on AUC measure can be found in [46].

*F-measure*. F-measure is a measure of a test's accuracy and often used in the field of information retrieval for measuring search, document classification, and query classification performance [47]. Both the precision 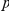 and the recall 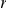 of the test are used to compute it. Here 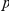 is the number of correct results divided by the number of all returned results while 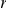 is the number of correct results divided by the number of results that should have been returned. The traditional F-measure is the harmonic mean of precision and recall:
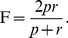
(15)The F-measure can be interpreted as a weighted average of the precision and recall. It ranges from 0 for highest dissimilarity to 1 for identical classifications.

## Results

### Evaluation on functionally related regulatory sequences

Regulatory sequence comparison plays an important role in the 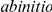 discovery of 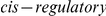 modules (CRMs) with a common function. If a set of co-regulated genes in a single species is given, we wish to find, in their upstream and downstream regions (henceforth called the ‘control regions’), the CRMs that mediate the common aspect of their expression profiles. The control regions may be tens of Kilobase long for each gene (especially for metazoan genomes), while the CRMs to be discovered are often only hundreds of base pair long. One must therefore search in the control regions for subsequences (the candidate CRMs) that share some functional similarity [5], [6].

The proposed 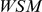 model is tested to evaluate if functionally related sequence pairs are scored better than unrelated pairs of sequences randomly chosen from the genome. In order to facilitate comparison, we choose following seven data sets published by Kantorovitz MR et al. [6]: FLY BLASTODERM (82 CRMs with expression in the blastoderm-stage embryo of the fruitfly, Drosophila melanogaster); FLY PNS [23 CRMs (average length 998 bp) driving expression in the peripheral nervous system in the fruitfly]; FLY TRACHEAL [9 CRMs (average length 1220 bp) involved in regulation of the tracheal system in the fruitfly]; FLYEYE [17 CRMs (average length 894 bp) expressing in the Drosophila eye ]; HUMAN MUSCLE [28 human CRMs (average length 450) regulating muscle specific gene expression]; HUMAN LIVER [9 CRMs (average length 201) driving expression specific to the human liver]; HUMAN HBB [17 CRMs (average length 453) regulating the HBB complex]. They are well studied by [5], [6], [48].

Experimental program is designed according to following settings: (1) A set of CRMs, known to regulate expression in the same tissue, is taken as the ‘positive’ set for each sequence in this set is the really 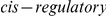 module, and a set of equally many randomly chosen noncoding sequences, with lengths matching the CRMs, is taken as the ‘negative’ set for each sequence in this set is the randomly chosen noncoding sequence not the really 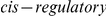 module. It would be interesting if we choose negative sequences from nearby regions of the known CRMs (positives), which will presumably have similar word distributions. Here, we chose seven noncoding data sets published by Kantorovitz MR et al. [6] to facilitate comparison with their results. (2) Each pair of sequences in the positive set is compared, and so is each pair in the negative set. (3) The evaluation procedure is based on a binary classification of each sequence pair, where 1 corresponds to the pairs from positive set, 0 corresponds to the pairs from negative set. Let 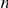 be the number of sequences in the positive set, all the pairs both from the positive and negative sets constitute a vector of length 2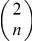. In addition, we can get a vector of length 2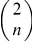 consisting of 1 and 0 as class labels. A perfect measure would completely separate the negative from the positive set. Of course, this does not happen in practice, and the classes are interspersed. The ROC curves permit to assess the level of accuracy of this separation without choosing any distance threshold for the separation point. In particular, the AUC will give us a unique number of the relative accuracy of each measure.

For comparison purpose, widely-used alignment tools were tested. These alignment tools include Needleman-Wunsch (global alignment) and Smith-Waterman (local alignment) raw scores, with no correction for statistical significance, using linear gap penalties or affine gap penalties, with a gap penalty of 2. We also implemented four word-based measures: Euclidean distance (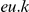) [27], Cosine distance (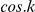) [31], Pearson's correlation coefficient (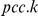) [32] and Kullback-Leibler discrepancy (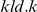) [29]. The performance of the proposed model was also compared with Markov models (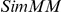 [2], composition vector (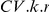 [33], [34]), 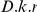 [35]) and mixed models (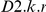 [49], 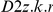 [6], 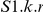 [5] and 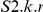 [5]). In addition to the alignment and statistical models, the improved composition vector (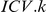) [36] was also tested. All statistical models based on the 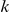-word distribution run with 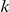 from 2 to 8. The 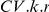, 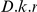, 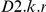, 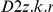, 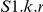, 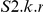 and 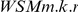 run with Markov order 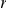 from 0 to 6 and the word length 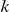 from 2 to 7. For each method, separate tests were performed with all combinations of parameter values, and the best combination was chosen to represent that score in the performance.

**Figure 1:**
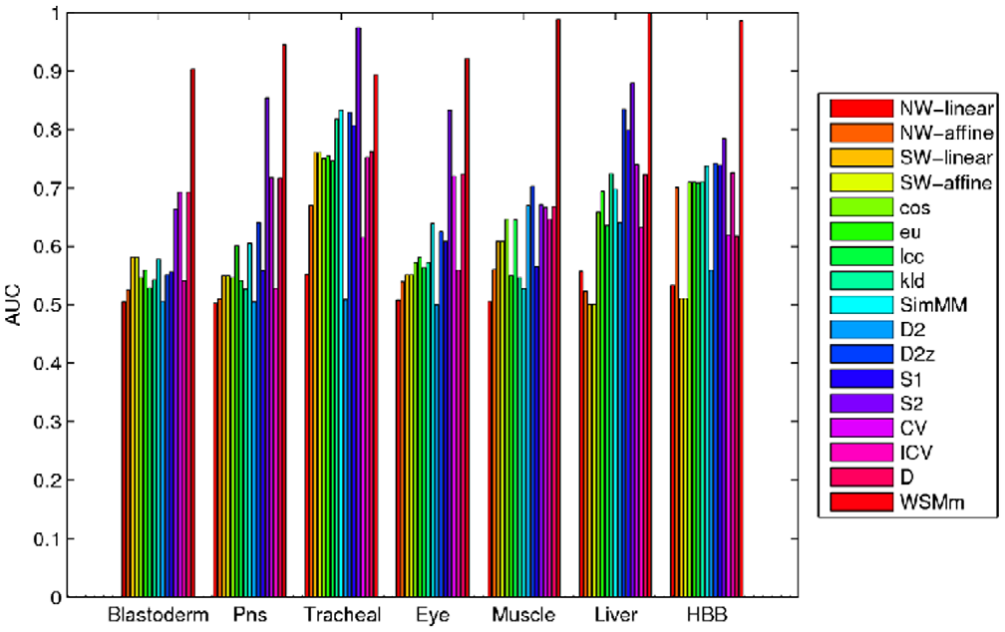
Comparison of AUCs of all models for detection of functionally related regulatory sequences. Comparison of AUCs of all models for detection of functionally related regulatory sequences. NW-linear and NW-affine denote Needleman-Wunsch (global alignment) raw scores, using linear gap penalties and affine gap penalties, respectively; SW-linear and SW-affine denote Smith-Waterman (local alignment) raw scores, using linear gap penalties and affine gap penalties, respectively; Word-based models are *eu*, *cos*, *pcc*, *kld*; Markov models are *SimM M*, *CV*, *D*; Mixed models are *D2*, *D2z*, *S1* and *S2*; Bernoulli model is *ICV*.

The AUCs for different methods are presented in Figure 1 and Table S1 in supplementary material. The first observation is that high accuracy of prediction can be achieved by the proposed measure 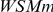. In the BLASTODERM experiment, the proposed measure 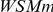 performs better than other alignment-based or alignment-free methods, with the area under ROC curve 0.9036. The next best method is the composition vector 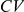. In the PNS experiment, the measure 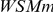 is better than all other measures, its area under ROC curve is 0.9456. In the TRACHEAL experiment, 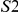 outperforms other measures, and its AUC is 0.975. It is followed by the measure 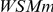. In the EYE experiment, the area under ROC curve of the measure 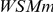 is 0.9216 , significantly better than that of other statistical methods. The next best measures is the measure 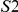. In the MUSCLE experiment, the measure 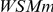 significantly outperforms other methods, and its area under ROC curve is 0.9892. It is followed by the 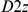. In LIVER experiments, the measure 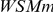 performs significantly better than other measures, with the area under ROC curve 0.9992. The next best measure is the measure 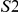. In HBB experiments, the measure 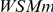 achieves the best performance, followed by the 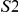. From the seven experiments, we can see that the proposed measure 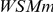 performs significantly better than other measures among six experiments, with AUC from 0.8935 to 0.9992.

### Human exons and introns classification

Numerous statistical algorithms have been proposed for exons and introns classification [50]–[53]. A basic assumption of these algorithms is that every exon in a genome should has some distinct sequence features or properties that can distinguish it from the surrounding regions, such as introns or intergenic regions. Competitive results have been obtained in the recognition of the exons and introns of prokaryotes gene, but the discrimination of the exons and introns in human is still a difficult problem because of their limited average length.

The secondary test of the proposed model is to discriminate the human exons and introns. These data sets were organized as follows: 1200 human exons and 1200 human introns are extracted from the human exon and intron data (http://bit.uq.edu.au/altExtron/for human exon and intron datasets), and they are randomly divided into four sets separately. The set of the exons is taken as the ‘positive’ set, and the set of the introns, is taken as the ‘negative’ set.

**Figure 2:**
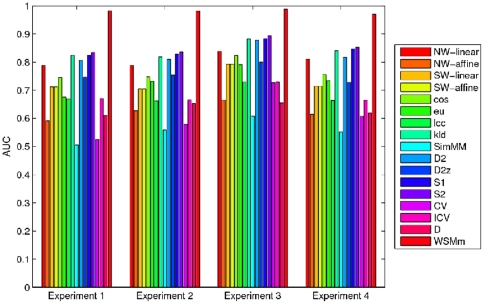
Comparison of AUCs of all models for classification of human exons and introns. Comparison of AUCs of all models for classification of human exons and introns. NW-linear and NW-affine denote Needleman-Wunsch (global alignment) raw scores, using linear gap penalties and affine gap penalties, respectively; SW-linear and SW-affine denote Smith-Waterman (local alignment) raw scores, using linear gap penalties and affine gap penalties, respectively; Word-based models are *eu*, *cos*, *pcc*, *kld*; Markov models are *SimM M*, *CV*, *D*; Mixed models are *D2*, *D2z*, *S1* and *S2*; Bernoulli model is *ICV*.

We took the previous evaluation procedure in this experiment, which make it easier to see effectiveness of various methods. The only difference lies in the parameter selection. Here all the models based on the 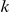-word frequency run with the word length 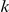 from 2 to 6, and the 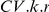, 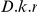, 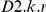, 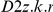, 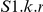, 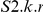 and 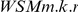 run with Markov order 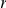 from 0 to 5 and the word length 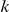 from 2 to 6. The AUCs for different methods are presented in Figure 2 and Table S2 in supplementary material.

In terms of the discriminative power, the proposed 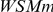 achieves the best performance compared to the existing methods, with AUC value ranging from 0.9704 to 0.9887 for the four classification tasks. These are excellent values, given that a perfect classification has an AUC score of 1, which indicates that the 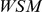 method is very effective to distinguish exons and introns in humans in despite of their limited average length.

### Clustering HEV genotype

Hepatitis E virus (HEV) is a major cause of enterically transmitted acute hepatitis in developing countries. HEV was classified recently as the sole member of the genus Hepevirus in the family Hepeviridae. Its genome consists of a single-stranded, positive-sense RNA of approximately 7.2 kb, with three partially overlapping open reading frames (ORFs: ORF1, ORF2, and ORF3). Although only one serotype has been identified to-date, HEV displays considerable genetic diversity. Based on the extensive full-length genomic variability noted among different strains, HEV has been classified into four major genotypes [54]. Here, a total of 48 full-length HEV genome sequences are retrieved from NCBI (http://www.ncbi.nlm.nih.gov/), which have been clustered into four genotypes [55]–[58]. Detail information on 48 full-length HEV genome sequences can be found in Table S3 in supplementary material.

This experiment aims at assessing how well the proposed model performs on identifying HEV genotype. In relation to the clustering literature [59], neighbor-joining [60] can be considered as a hierarchical method. It is chosen to clustering HEV genotypes, which is implemented in BioPerl [61]. As HEV genotypes is a 4-classification problem rather than one, F-measure was used to capture overall performance on HEV genotypes. To evaluate a clustering problem using the F-measure, we need to select a gold standard [59]. Here, the traditional classification was used as the gold standard [54].

**Figure 3:**
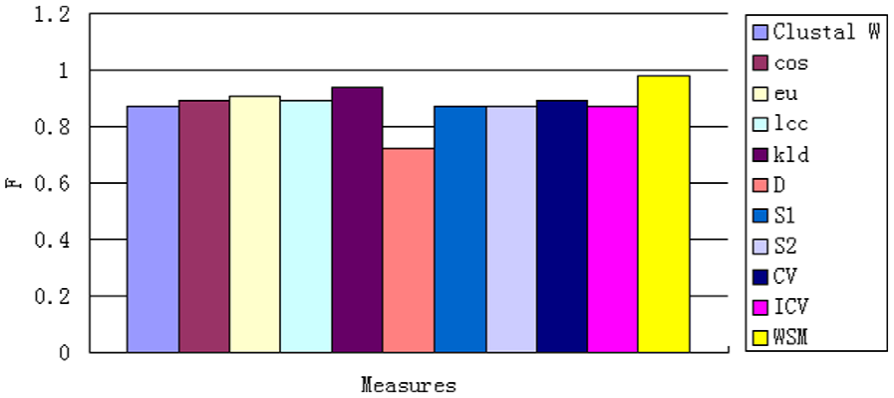
Comparison of F-measures of all models for classification of HEV genotypes. Comparison of F-measures of all models for classification of HEV genotypes. NW-linear and NW-affine denote Needleman-Wunsch (global alignment) raw scores, using linear gap penalties and affine gap penalties, respectively; SW-linear and SW-affine denote Smith-Waterman (local alignment) raw scores, using linear gap penalties and affine gap penalties, respectively.

In addition to the proposed method, four other typical methods were used for comparison. The used alignment-based method is Clustal W rather than Needleman-Wunsch (global alignment) or Smith-Waterman (local alignment) raw scores, because the length of genome of the HEV is approximately 7.2 kb that is difficult to handle by dynamic algorithm. The measures 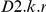 and 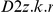 were not evaluated as they do not satisfy the identity condition. All statistical models based on the 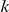-word distribution run with 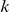 from 2 to 8. The 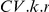, 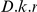, 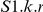, 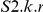 and 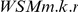 run Markov order 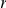 from 0 to 7 and the word length 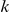 from 2 to 8. Figure 3 reports the F-measure for all methods on the 48 HEV genomes data set, and more details can be found in Table S4 in supplementary material.

Figure 3 shows that the proposed 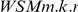 performs better than the other alignment-based or alignment-free methods, with the F-measure 0.9791. This result is consistent with the above results, and we attribute this to the combination of both the words' overlapping structures and words' background information.

### Influence of the overlapping structures of the words

**Figure 4:**
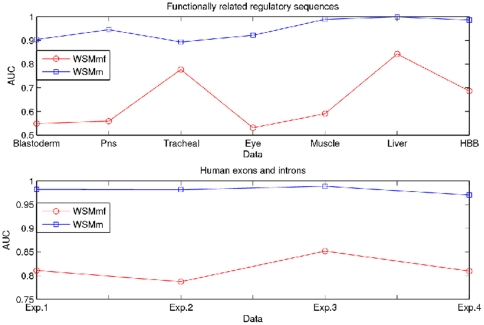
Comparison of AUCs of the measures *WSMm* and *WSMmf*. From top down, comparison of AUCs of the measures *WSMm* and *WSMmf* for predicting functionally related regulatory sequences and classifying human exons and introns.

For a better understanding of the proposed method, an evaluation of the word overlapping structures in biological sequences was performed. A measure, 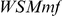, which is similar to 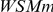 but defined based on the 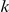-word frequencies is defined as follows:
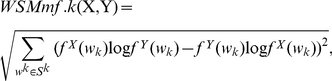
(16)where 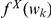 and 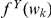 are the frequencies of the 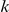-words in the biological sequences 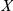 and 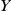. The only difference between the measures 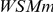 and 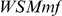 is that the overlapping word is considered in the former. Therefore the improvement of the measure 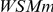 can be solely attributed to the overlapping words involved. The AUCs for the measures 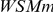 and 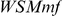 are presented in Figure 4.

We observe that the measure 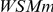 significantly outperforms the measure 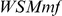 among all the experiments. For functionally related regulatory sequences, classification accuracies of the proposed measure 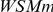 are as high as 0.8935
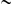
0.9992 in comparison to 0.5308
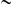
0.8426 with the measure 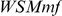. For human exons and introns classification, the accuracies achieved by the proposed measure 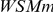 is 0.9704
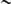
0.9887, while the measure 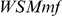 only reaches 0.7871
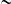
0.8518. These results strongly demonstrate that incorporation of the overlapping words information consistently improves both efficiency and effectiveness of the sequence comparison.

### Influence of the estimated word variance

Another feature of the proposed measure 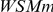 is that the word variance is estimated upon observed biological sequences without assuming the bases occur randomly with equal chance. To show the efficiency of the estimated word variances, we compared the proposed measure 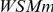 with another statistical measure, 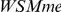, defined as follows:
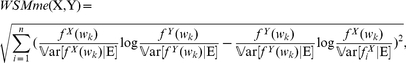
(17)where
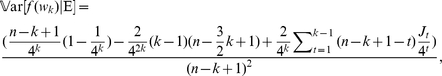
and E denotes a known word distribution in which the four bases A, C, T, and G occur randomly with equal chance [36], 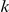 is the length of the words in biological sequences, and 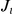 is an indicator function, equal to 1 if 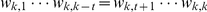 and equal to 0 otherwise, for 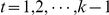.

**Figure 5:**
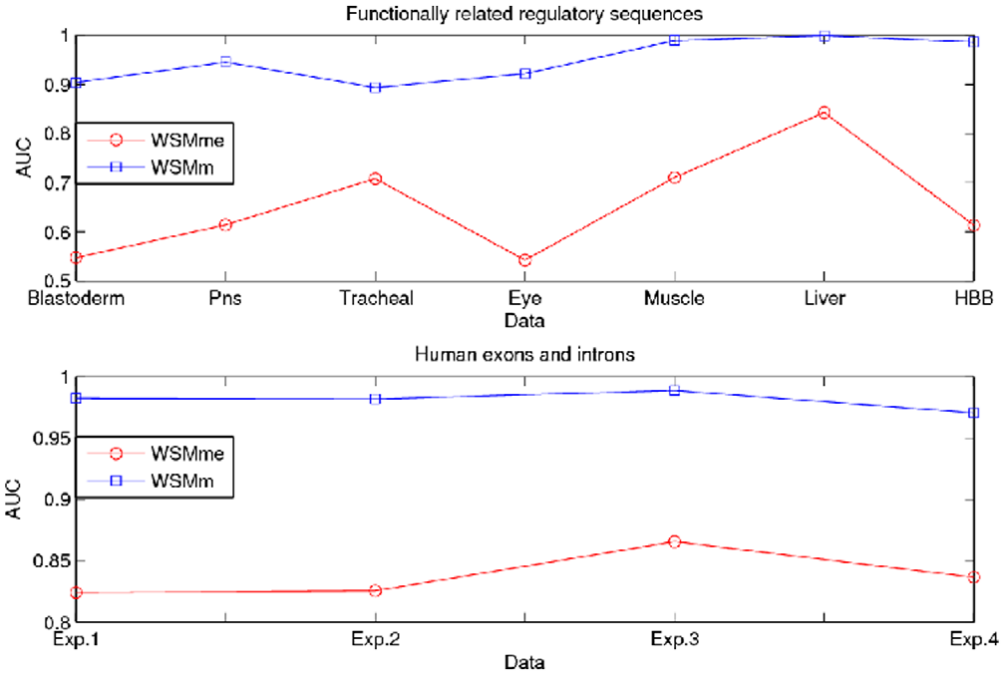
Comparison of AUCs of the measures *WSMm* and *WSMme*. From top down, comparison of AUCs of the measures *WSMm* and *WSMme* for predicting functionally related regulatory sequences and classifying human exons and introns.

The 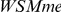 assumes that the four bases A, C, T, and G occur randomly with equal chance, while the proposed measure 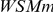 estimates the word variances according to the observed biological sequences. The comparison between the measures 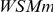 and 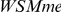 should suggest the influence of the estimated word variance. The AUCs for the measures 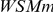 and 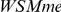 are listed in Figure 5.

In all cases, the classification of the proposed measure 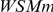 is more accurate than that of the measure 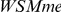. For example, by using the estimated word variance, the proposed measure 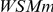 detects the functionally related regulatory sequences with accuracies of 0.8935
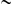
0.9992, while the measure 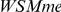 only detects 0.542
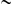
0.8426; in the case of discrimination of human exons and introns, 0.9704
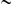
0.9887 for the measure 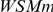 contrasts with 0.8241
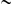
0.8656 for the measure 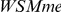. These results demonstrate that estimating variances from the observed sequences could be more promising to improve the biological sequence comparison because it helps the measure 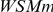 to adjust the background information according to the word distribution.

## Discussion

This paper proposed an efficient statistical method for biological sequence comparison, which integrates both the overlapping structures and background information of the words in biological sequences. It compares biological sequence by taking advantage of the tendency of the 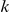-word conservation. In the application, the proposed method treats the word appearing at a given position as a random variable, estimates the word variance according to the observed sequence, and therefore maximizes the impact of the overlapping structures and background information of the words in sequence. A similar idea was proposed in our previous measures 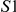 and 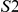, but as shown in our experiments, the proposed measure 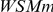 performs significantly better which suggests that the overlapping structures and background information of the words should be included in word-based statistical methods to improve biological sequence comparison.

The proposed method originates from the existing methods but different from them in several key aspects. Blaisdell, Wu et al. and Stuart et al. [27], [29], [31] developed popular sequence comparison methods where similarity/dissimilarity score depends on the measure under the frequency vector of the 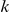-words in biological sequence. However, they did not use the background information of 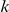-words for sequence comparison, and the probability of the 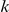-words under these models is estimated by the occurrences of the 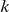-words. Pham and Zuegg [2] also proposed ways to improve biological sequence comparison, but their model is different from ours in that the appearance of the 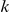-words are modeled by a Markov model, whose parameters are independent of the 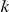-word distribution in biological sequence. We developed a Markov plus 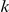-word distribution model [5], based on the idea of adding k-word distribution in sequence to Markov model directly. The way of treating sequence comparison is also different from the proposed method: no information about the overlapping structure of a word in biological sequence was considered in our previous mixed model. Lu et al. [36] found some statistical problems associated with composition vector (CV) [33], [34] and proposed an improved composition vector (ICV) method. Their study assumes that the four bases A, C, T, and G occur randomly with equal chance and derives the expected count of a 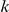-word and the count variance in a given sequence 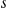 based upon this simple assumption. In other words, the word distribution is assumed to be known a priori. But, in most cases the word distribution is usually unknown, and therefore the application of ICV method is very limited in practice. Most importantly, this research demonstrated that integration the overlapping structure of a word with the estimated background information of the words according to the observed sequences is essential to improve biological sequence comparison. In addition, among tree kinds of the experiments, the length of biological sequence varies from 201 (HUMAN LIVER [9 CRMs (average length 201) driving expression specific to the human liver]) to 7.2 kb (the genome of HEV consists of a single-stranded, positive-sense RNA of approximately 7.2 kb). The proposed method achieved the best performance among all the experiments, which indicates that its performance is not influenced by the sequence length. As for the computational efficiency, because the 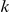-words in biological sequence are considered in the definition of the statistical measure 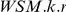, its computational efficiency is the same as that of existing methods based on the word-based models [2], [5], [27]–[29], [31], [33], [34], [36].

One major limitation of the proposed method is that different 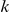-words are assumed to be independent under Bernoulli and Markov model which is not always met in practice, and their influence should be taken into consideration. One consequence of our simplification is that the correlations between different 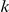-words are ignored and only the same k-word variances are accounted for. A better model should reflect the data covariance structure. Despite of this simplification, we found that the proposed statistical measure essentially improves biological sequence comparison.

## Supporting Information

Table S1 AUCs obtained from all the models for detection of functionally related regulatory sequences.
(DOC)

Table S2 AUCs obtained from all the models for classification of human exons and introns.
(DOC)

Table S3 Abbreviation for the strains, accession number, nucleotide length, genotype, and country for each of the 48 complete HEV genomes.
(DOC)

Table S4 F-measures obtained from all the models for classification of HEV genotypes.
(PDF)
