# Supplementary material for: Integrating Overlapping Structures and Background Information of Words Significantly Improves Biological Sequence Comparison
Source: PLoS One. 2011 Nov 10;6(11):e26779. doi: 10.1371/journal.pone.0026779 (PMC3213098; doi:10.1371/journal.pone.0026779)
Supplement: Table S1 — AUCs obtained from all the models for detection of functionally related regulatory sequences. (DOC) [file pone.0026779.s001.doc]

Table S1. AUCs obtained from all the models for detection of functionally related regulatory sequences

| Blastoderm | | Pns | | Tracheal | |
| --- | --- | --- | --- | --- | --- |
| Methods | AUC | Methods | AUC | Methods | AUC |
| NW-linear | 0.505 | NW-linear | 0.503 | NW-linear | 0.553 |
| NW-affine | 0.526 | NW-affine | 0.51 | NW-affine | 0.671 |
| SW-linear | 0.582 | SW-linear | 0.55 | SW-linear | 0.761 |
| SW-affine | 0.582 | SW-affine | 0.55 | SW-affine | 0.761 |
| Cos.2 | 0.547 | Cos.4 | 0.547 | cos.2 | 0.75 |
| Eu.2 | 0.56 | Eu.8 | 0.601 | eu.2 | 0.755 |
| Lcc.2 | 0.529 | Lcc.4 | 0.542 | lcc.2 | 0.746 |
| Kld.2 | 0.543 | Kld.2 | 0.527 | kld.2 | 0.818 |
| Simm | 0.579 | Simm | 0.605 | Simm | 0.833 |
| D2.2.0 | 0.506 | D2.2.0 | 0.505 | D2.2.1 | 0.509 |
| D2z.2.1 | 0.552 | D2z.3.2 | 0.641 | D2z.4.0 | 0.829 |
| D.3 | 0.693 | D.3 | 0.717 | D.3 | 0.762 |
| S1.3.2 | 0.557 | S1.2.0 | 0.559 | S1.3.2 | 0.807 |
| S2.7.2 | 0.664 | S2.2.0 | 0.854 | S2.8.2 | 0.975 |
| CV.3.2 | 0.6932 | CV.3.2 | 0.718 | CV.5.4 | 0.6157 |
| ICV.2 | 0.5417 | ICV.6 | 0.5286 | ICV.2 | 0.7523 |
| WSMm.3.2 | 0.9036 | WSMm.3.2 | 0.9456 | WSMm.6.0 | 0.8935 |
|  | | | | | |
| Eye | | Muscle | | Liver | |
| Methods | AUC | Methods | AUC | Methods | AUC |
| NW-linear | 0.508 | NW-linear | 0.506 | NW-linear | 0.558 |
| NW-affine | 0.54 | NW-affine | 0.561 | NW-affine | 0.523 |
| SW-linear | 0.552 | SW-linear | 0.609 | SW-linear | 0.501 |
| SW-affine | 0.552 | SW-affine | 0.609 | SW-affine | 0.501 |
| cos.3 | 0.573 | cos.8 | 0.647 | cos.2 | 0.659 |
| eu.3 | 0.582 | eu.2 | 0.55 | eu.2 | 0.695 |
| lcc.3 | 0.564 | lcc.7 | 0.646 | lcc.7 | 0.636 |
| kld.2 | 0.573 | kld.2 | 0.547 | kld.2 | 0.725 |
| Simm | 0.64 | Simm | 0.528 | Simm | 0.698 |
| D2.8.0 | 0.5 | D2.8.2 | 0.671 | D2.7.1 | 0.641 |
| D2z.4.0 | 0.626 | D2z.2.0 | 0.703 | D2z.2.0 | 0.835 |
| D.3 | 0.724 | D.3 | 0.669 | D.3 | 0.723 |
| S1.3.2 | 0.609 | S1.3.2 | 0.566 | S1.8.1 | 0.799 |
| S2.8.2 | 0.833 | S2.8.0 | 0.672 | S2.5.1 | 0.88 |
| CV.3.2 | 0.720 | CV.3.2 | 0.667 | CV.3.2 | 0.74 |
| ICV.3 | 0.560 | ICV.7 | 0.647 | ICV.7 | 0.633 |
| WSMm.3.2 | 0.922 | WSMm.3.2 | 0.989 | WSMm.3.2 | 0.999 |

| HBB | |  | |  | |
| --- | --- | --- | --- | --- | --- |
| Methods | AUC |  |  |  |  |
| NW-linear | 0.534 |  |  |  |  |
| NW-affine | 0.701 |  |  |  |  |
| SW-linear | 0.511 |  |  |  |  |
| SW-affine | 0.511 |  |  |  |  |
| cos.2 | 0.71 |  |  |  |  |
| eu.2 | 0.71 |  |  |  |  |
| lcc.2 | 0.709 |  |  |  |  |
| kld.2 | 0.711 |  |  |  |  |
| Simm | 0.738 |  |  |  |  |
| D2.5.1 | 0.56 |  |  |  |  |
| D2z.3.0 | 0.742 |  |  |  |  |
| D.3 | 0.618 |  |  |  |  |
| S1.3.2 | 0.739 |  |  |  |  |
| S2.2.0 | 0.785 |  |  |  |  |
| CV.3.2 | 0.620 |  |  |  |  |
| ICV.2 | 0.727 |  |  |  |  |
| WSMm.3.2 | 0.987 |  |  |  |  |
