# Supplementary material for: Integrating Overlapping Structures and Background Information of Words Significantly Improves Biological Sequence Comparison
Source: PLoS One. 2011 Nov 10;6(11):e26779. doi: 10.1371/journal.pone.0026779 (PMC3213098; doi:10.1371/journal.pone.0026779)
Supplement: Table S2 — AUCs obtained from all the models for classification of human exons and introns. (DOC) [file pone.0026779.s002.doc]

Table S2. AUCs obtained from all the models for classification of human exons and introns

| Experiment 1 | | Experiment 2 | | Experiment 3 | | Experiment 4 | |
| --- | --- | --- | --- | --- | --- | --- | --- |
| Methods | AUC | Methods | AUC | Methods | AUC | Methods | AUC |
| NW-linear | 0.789 | NW-linear | 0.789 | NW-linear | 0.839 | NW-linear | 0.811 |
| NW-affine | 0.591 | NW-affine | 0.627 | NW-affine | 0.665 | NW-affine | 0.614 |
| SW-linear | 0.712 | SW-linear | 0.704 | SW-linear | 0.793 | SW-linear | 0.714 |
| SW-affine | 0.712 | SW-affine | 0.704 | SW-affine | 0.793 | SW-affine | 0.714 |
| cos.6 | 0.745 | cos.5 | 0.748 | cos.6 | 0.824 | cos.6 | 0.756 |
| eu.6 | 0.676 | eu.6 | 0.731 | eu.6 | 0.791 | eu.6 | 0.733 |
| lcc.6 | 0.669 | lcc.6 | 0.662 | lcc.5 | 0.729 | lcc.5 | 0.663 |
| kld.5 | 0.824 | kld.5 | 0.819 | kld.5 | 0.882 | kld.5 | 0.841 |
| Simm | 0.505 | Simm | 0.559 | Simm | 0.608 | Simm | 0.552 |
| D2.8.0 | 0.807 | D2.3.0 | 0.810 | D2.4.0 | 0.878 | D2.3.0 | 0.818 |
| D2z.3.0 | 0.746 | D2z.3.0 | 0.754 | D2z.3.0 | 0.800 | D2z.3.0 | 0.728 |
| D.3 | 0.610 | D.3 | 0.653 | D.3 | 0.655 | D.3 | 0.619 |
| S1.3.1 | 0.824 | S1.4.1 | 0.829 | S1.7.1 | 0.884 | S1.4.1 | 0.848 |
| S2.3.1 | 0.835 | S2.6.1 | 0.836 | S2.6.1 | 0.894 | S2.7.1 | 0.853 |
| CV.3.2 | 0.525 | CV.3.2 | 0.579 | CV.6.5 | 0.726 | CV.3.2 | 0.607 |
| ICV.5 | 0.670 | ICV.5 | 0.666 | ICV.5 | 0.730 | ICV.5 | 0.665 |
| WSMm.3.2 | 0.982 | WSMm.3.2 | 0.982 | WSMm.3.2 | 0.989 | WSMm.3.2 | 0.970 |
