# Supplementary material for: Integrating Overlapping Structures and Background Information of Words Significantly Improves Biological Sequence Comparison
Source: PLoS One. 2011 Nov 10;6(11):e26779. doi: 10.1371/journal.pone.0026779 (PMC3213098; doi:10.1371/journal.pone.0026779)
Supplement: Table S3 — Abbreviation for the strains, accession number, nucleotide length, genotype, and country for each of the 48 complete HEV genomes. (DOC) [file pone.0026779.s003.doc]

| Method | AUC |
| --- | --- |
| Clustal W | 0.8696 |
| Cos.8 | 0.891 |
| Eu.5 | 0.9082 |
| Lcc.7 | 0.891 |
| Kld.7 | 0.9429 |
| Simm | 0.7231 |
| D.6 | 0.965 |
| S1.5.4 | 0.8696 |
| S2.5.4 | 0.8696 |
| CV.7.6 | 0.891 |
| ICV.7.6 | 0.8696 |
| WSMm.6.5 | 0.9791 |

Table S3. F-measure obtained from all the models for classification of HEV genotypes
