# Supplementary material for: Integrating Overlapping Structures and Background Information of Words Significantly Improves Biological Sequence Comparison
Source: PLoS One. 2011 Nov 10;6(11):e26779. doi: 10.1371/journal.pone.0026779 (PMC3213098; doi:10.1371/journal.pone.0026779)
Supplement: Table S4 — F-measures obtained from all the models for classification of HEV genotypes. (PDF) [file pone.0026779.s004.pdf]

Table S4

Abbreviation for the strains, accession number, nucleotide length, genotype, and country for each of the 48 complete HEV genomes

No.	Strain name	Accession No.	Nucleotide length	Genotype	Country
1	B1 (Bur-82)	M73218	7207	I	Burma (Rangoon)
2	B2 (Bur-86)	D10330	7194	I	Burma (Rangoon)
3	I2 [Mad-93]	X99441	7194	I	India (Madras)
4	I3	AF076239	7194	I	India (Hyderabad)
5	Np1 (TK15/92)	AF051830	7199	I	Nepal (Kathamandu)
6	P2 [Abb-2B]	AF185822	7143	I	Pakistan (Abbottabad)
7	Yam-67	AF459438	7206	I	India (Yamuna Nagar)
8	C1 (CHT-88)	D11092	7207	I	China (Xinjiang, Hetian)
9	C2 (KS2-87)	L25595	7221	I	China (Xinjiang, Kashi)
10	C3 (CHT-87)	L08816	7176	I	China (Xinjiang, Hetian)
11	C4 (Uigh179)	D11093	7194	I	China (Xinjiang, Uighur)
12	China Hebei	M94177	7200	I	China (Hebei)
13	P1 (Sar-55)	M80581	7138	I	Pakistan (Sargodha)
14	I1 (FHF)	X98292	7202	I	India
15	Morocco	AY230202	7212	I	Morocco
16	T3	AY204877	7170	I	Chad
17	M1	M74506	7180	II	Mexico (Telixtac)
18	HE-JA10	AB089824	7262	III	Japan (Tokyo)
19	JKN-Sap	AB074918	7256	III	Japan (Sapporo)
20	JMY-HAW	AB074920	7240	III	Japan (Sapporo)
21	swUS1	AF082843	7207	III	USA
22	US1	AF060668	7202	III	USA (Minnesota)
23	US2	AF060669	7277	III	USA (Tennessee)
24	JBOAR1-Hyo04	AB189070	7247	III	Japan (Hyogo)
25	JDEER-Hyo03L	AB189071	7230	III	Japan (Hyogo)
26	JJT-KAN	AB091394	7218	III	Japan (Kanagawa)
27	JMO-Hyo03L	AB189072	7180	III	Japan (Hyogo)
28	JRA1	AP003430	7230	III	Japan (Tokyo)
29	JSO-Hyo03L	AB189073	7180	III	Japan (Tokyo)
30	JTH-Hyo03L	AB189074	7180	III	Japan (Tokyo)
31	JYO-Hyo03L	AB189075	7180	III	Japan (Tokyo)
32	swJ570	AB073912	7257	III	Japan (Tochigi)
33	Kyrgyz	AF455784	7239	III	Kyrgyzstan
34	Arkell	AY115488	7255	III	Canada (Ontario, Guelph)
35	HE-JA1	AB097812	7258	IV	Japan (Hokkaido)
36	HE-JK4	AB099347	7250	IV	Japan (Tochigi)
37	HE-JI4	AB080575	7186	IV	Japan (Tochigi)
38	JAK-Sai	AB074915	7236	IV	Japan (Saitama)
39	JKK-Sap	AB074917	7235	IV	Japan (Sapporo)
40	JSM-Sap95	AB161717	7202	IV	Japan (Hokkaido)
41	JSN-Sap-FH	AB091395	7234	IV	Japan (Hokkaido)
42	JSN-Sap-FH02C	AB200239	7251	IV	Japan (Hokkaido)
43	JTS-Sap02	AB161718	7202	IV	Japan (Hokkaido)
44	JYW-Sap02	AB161719	7202	IV	Japan (Hokkaido)
45	swJ13-1	AB097811	7258	IV	Japan (Hokkaido)
46	swCH25	AY594199	7270	IV	China (Uighur)
47	T1	AJ272108	7232	IV	China (Beijing)
48	CCC220	AB108537	7193	IV	China (Changchun)
